# Perceptions and intentions relating to seeking help for depression among medical undergraduates in Sri Lanka: a cross-sectional comparison with non-medical undergraduates

**DOI:** 10.1186/s12909-015-0453-8

**Published:** 2015-09-29

**Authors:** Santushi D. Amarasuriya, Anthony F. Jorm, Nicola J. Reavley

**Affiliations:** 1 Behavioural Sciences Stream, Faculty of Medicine, University of Colombo, PO Box 271, 25, Kynsey Road, Colombo 8, Sri Lanka; 2 Centre for Mental Health, Melbourne School of Population and Global Health, University of Melbourne, 207 Bouverie Street, Victoria, 3010 Australia

**Keywords:** Medical, Undergraduate, Depression, Help-seeking, Attitudes, Beliefs, Perceptions, Intentions, Sri Lanka

## Abstract

**Background:**

This study attempts to understand whether medical undergraduates in Sri Lanka would seek help for depression. This was done by examining their perceptions and intentions relating to seeking help for depression, using the responses of non-medical undergraduates as the baseline for comparison.

**Method:**

Medical (*n* = 620) and non-medical undergraduates (*n* = 4050) at the University of Colombo responded to a questionnaire which included a vignette about a depressed undergraduate, a depression measure, an open-ended question examining their intentions to seek help if affected by the problem described in the vignette, and scales examining their perceptions about the helpfulness of various help-seeking options for dealing with the problem. The latter items were also administered among mental health professionals to assess *expert opinion* on dealing with depression. Logistic regression models were used to examine if medical undergraduates differed from non-medical undergraduates in their rates of depression, help-seeking perceptions and help-seeking intentions. These models were also used to examine if being depressed was associated with differences in the help-seeking perceptions and intentions of medical undergraduates.

**Results:**

Medical and non-medical undergraduates did not differ in their odds of being depressed. Overall, the medical undergraduates were more likely to appraise professional help positively. However, they did not differ from non-medical undergraduates in relation to their intentions to seek such help if affected by the problem personally. They were also more likely to indicate their intentions to seek help from parents and family. Furthermore, medical undergraduates who screened positive for Major Depression were less likely to appraise some of the recommended professional and informal help-seeking options as being ‘helpful’, with only 50 % considering that it was ‘unhelpful’ to deal with the problem alone. There was also no difference in their help-seeking intentions as compared to those screening negative for Major Depression.

**Discussion and Conclusions:**

Although medical training seems to be associated with better help-seeking beliefs, interventions are needed to improve these medical undergraduates’ intentions to personally seek professional help for depression. It is concerning that medical undergraduates who are depressed might be less likely to consider it beneficial to seek help and instead, deal with the problem alone.

**Electronic supplementary material:**

The online version of this article (doi:10.1186/s12909-015-0453-8) contains supplementary material, which is available to authorized users.

## Background

Several studies indicate that there is a high prevalence of depression among undergraduates [1, 2]. Studies also highlight this among medical undergraduates [3–7]. However, there is conflicting evidence as to whether the rate of depression differs between medical and non-medical undergraduates, and if so, in which group it is higher [4, 8]. Regardless, it is necessary to examine if those in medical schools are depressed. While undergraduate medical training involves an inherently strenuous course, studies from Sweden and Norway indicate that depression and mental health problems among medical undergraduates might be associated with the stress they experience due to the medical course and the university experience [5, 9]. Furthermore, medical undergraduates suffering from such mental health problems are shown to be at risk of facing academic difficulties and potential drop-out from medical school [6, 10] and to also suffer from mental health problems in their later careers as doctors [11, 12]. Hence, medical undergraduates affected by such problems must receive the necessary assistance.

Given that medical undergraduates have a greater opportunity for exposure to mental health information as compared to non-medical undergraduates, it would be expected that they possess higher levels of mental health literacy, i.e., knowledge relevant to recognition, treatment and prevention of mental disorders [13]. Indeed, studies do show that being a medical student, as well as more years of medical training, are associated with higher levels of mental health literacy [14, 15]. In light of such findings it would also be expected that medical undergraduates who are suffering from mental health problems would seek the necessary professional assistance.

Contrary to this expectation, studies show that only a low proportion of these medical undergraduates actually seek such help [7, 9, 16–19]. Instead, they show preference to seek help from informal sources, such as friends and family [18, 20–23]. This trend has also been seen among doctors and psychiatrists [24–27]. Some of the reasons that medical undergraduates cite for their reluctance to seek mental health services, are their fear that disclosure of mental health problems would impact their academic record and affect their future medical career, concerns about breaches in confidentiality, fear of stigma and discrimination by others in their profession, and embarrassment about succumbing to such problems [3, 7, 18–20, 28]. Studies also indicate that medical undergraduates who are depressed are more likely to be reluctant to disclose their problems to professionals and to perceive others to hold stigma about depression [3, 29].

Given such findings, it is necessary to ascertain if medical undergraduates would seek the appropriate help for their mental health problems. Such an examination becomes especially important in developing countries such as Sri Lanka, where the number of mental health professionals in the country is low [30], and where undergraduates might have access to only a limited number of these professionals, with many affiliated to the university as lecturers or functioning as clinical teachers. Hence, these undergraduates might show greater reluctance to seek assistance for their mental health problems. Studies also show that medical undergraduates in Sri Lanka hold several stigmatising attitudes about the mentally ill [31]. In turn, these attitudes could negatively influence their help-seeking practices [32, 33]. This further stresses the need for understanding whether these undergraduates would seek the help they need. However, such research is currently unavailable in Sri Lanka. Therefore, this study focused on understanding whether these undergraduates would seek assistance for depression, a disorder common among medical undergraduates. Depression is also the mental disorder that leads to most premature deaths and disability in Sri Lanka [34], and among those between 20–29 years, the age range of most undergraduates [35]. Hence, it was considered appropriate to focus on help-seeking for depression, in this study.

Theories of help-seeking present *help-seeking beliefs* and *intentions* as common constructs within the help-seeking process, both considered to be indicators of help-seeking behaviours [36]. Many studies examining the *help-seeking beliefs* of undergraduates have examined their perceptions about the helpfulness of various help-seeking options [37–40]. Such perceptions could be considered to reflect knowledge and attitudes about seeking help for the relevant problem, and to also influence help-seeking intentions and actions [41, 42]. Hence, the help-seeking perceptions of the undergraduates were examined. However, there is also evidence that positive attitudes about help-seeking, such as getting help from mental health professionals, might not always translate into related actions [43, 44]. Hence, we found it necessary to also examine these undergraduates’ *intentions* to seek help as these might be stronger indicators of their actual help-seeking actions [45]. The assessment of both the help-seeking perceptions and intentions of these undergraduates was considered to provide a more comprehensive understanding of their inclination to seek help for depression.

As it was important to examine the responses of the medical undergraduates within the context of being university students in general, the related responses of non-medical undergraduates were used for comparison. Given the lack of professional mental health services in the country, it was also necessary to use a culturally-relevant benchmark for assessing these undergraduates’ help-seeking perceptions, regarding the *helpfulness* of various options of help. Therefore, the related responses of Sri Lankan mental health professionals were used as an indicator of *expert opinion* about help-seeking. We attempted to answer the following research questions: (1) Is the prevalence of depression among medical undergraduates different from that amongst non-medical undergraduates?; (2) Are the help-seeking perceptions of medical undergraduates different from those of non-medical undergraduates? (with expert opinion also used as a benchmark for comparison); (3) Are the help-seeking intentions of medical undergraduates different from those of non-medical undergraduates?; (4) Are the help-seeking perceptions of medical undergraduates who are depressed different from those who are not?; (5) Are the help-seeking intentions of medical undergraduates who are depressed different from those who are not?

## Method

### Setting

This cross-sectional study was a part of a larger Depression Literacy Survey done among undergraduates at the University of Colombo, one of the largest universities in Sri Lanka [46]. At present there are no formalised mental health services in the university. Academics or other staff appointed to the role of *student counsellor* act as contact points for undergraduates who need assistance with various problems. Those requiring mental health assistance are referred by them to academic staff, who are mental health professionals or have mental health training, or to mental health professionals affiliated to the university.

### Participants

Data was collected from undergraduates in all five years of study at the Medical Faculty. As previous studies have highlighted differences in mental health knowledge and practices of undergraduates among different non-medical disciplines [47], this study involved the participation of undergraduates from all other faculties of the University of Colombo. This included undergraduates from all years of study (four years being the maximum) from the Faculties of Arts, Law, Management and Finance, Science, and the School of Computing, an affiliated institute of the University. Although data was not collected from the Faculty of Education, second and third year students of this faculty attend lectures at the Faculty of Arts and hence were represented during data collection.

### Measure

The Depression Literacy Survey, which this study was a part of, utilised a dual-language questionnaire, with English-Sinhala and English-Tamil versions, with participants able to request their preferred version. While mental health literacy measures used in similar studies provided the basic template for this questionnaire [13, 37], it underwent several stages of adaptation prior to its use. We only describe here the sections of the questionnaire which are relevant to the present study (see Additional file 1 for questionnaire).

Subsequent to a section for demographic information, participants were presented with a vignette of an undergraduate named “Z”, described to be of their same age and gender, exhibiting symptoms of Major Depression as per the Diagnostic and Statistical Manual of Mental Disorders-IV [48].

They were then presented with open-ended questions asking what they thought was wrong with ‘Z’ and what they would do if personally affected by the problem (help-seeking intentions).

Participants were also presented with scales assessing their perceptions about the helpfulness of a range of help-seeking options to help “Z” to deal with this problem (rated as ‘very helpful’, ‘fairly helpful’, ‘neither helpful nor unhelpful’, ‘fairly unhelpful’, ‘very unhelpful’, ‘don’t know’). These scales not only included professional help-seeking options but also the more informal options that the undergraduates could seek assistance from. They also included items relevant to both the Sri Lankan context and the services relevant to the study site. Subsequent to cultural adaptation of the measure, this section consisted of a total of 50 items.

The questionnaire also included the Patient Health Questionnaire-9 (PHQ-9) for screening depression along with its Sinhala/Tamil validated version [49].

### Procedure

#### Administration of questionnaire

The questionnaires were administered during lectures common to each year of study at the Medical and other Faculties/Schools. However, as it was difficult to identify any common lectures at the Faculty of Arts due to the varied subject combinations, lectures with the largest student cohorts were approached. The questionnaires were distributed to all students who were present at each of the lectures. During distribution, the potential participants were given a brief introduction to the study. In most instances this was given by the first author (SDA). In her absence, an introductory statement was read out by the lecturer present. The undergraduates were also informed that participation was voluntary. The potential participants were then referred to the participant information sheet which provided more details about the study, including that if a filled questionnaire was returned, this implied their consent to participate in the study. Participants completed the composite Depression Literacy Survey in approximately 20 min.

### Expert opinion about help-seeking (to assess undergraduates’ help-seeking perceptions)

The scales examining the undergraduates’ perceptions about the *helpfulness* of different help-seeking options were also administered among mental health professionals (psychiatrists and clinical psychologists) in Sri Lanka. This was carried out via an online survey and 37 valid responses were obtained (being 36 % of the total population of mental health professionals identified via their respective professional/registration bodies). Help-seeking options that were endorsed as ‘helpful’ by ≥ 75 % of these *mental health experts* (‘very helpful’ or ‘fairly helpful’) were considered as the recommended options for dealing with depression for this study population. Options rated as ‘unhelpful’ by ≥ 75 % of these experts (‘very unhelpful’ or ‘fairly unhelpful’) were used to assess the unhelpful (or inappropriate) help-seeking beliefs of this population. Out of the 50 help-seeking options presented, nine professional/formal and eleven informal options were recommended as ‘helpful’, with the latter including self-help strategies. Furthermore, three options were identified as ‘unhelpful’, with two of these being self-help strategies (see Additional file 2 for the list of options identified as ‘helpful’ and ‘unhelpful’ by the experts).

As the study aimed to examine the undergraduates’ perceptions about help-seeking, only data for the options associated with seeking the assistance of others were used. This involved nine professional/formal and four informal help-seeking options that were recommended as being ‘helpful’ and one option that was identified as being ‘unhelpful’ by the experts (See Additional file 2).

The undergraduates’ ratings for these help-seeking options were recoded, to examine if they perceived them as being ‘helpful’ (‘very helpful’ or ‘fairly helpful’) or ‘unhelpful’ (‘very unhelpful’ or ‘fairly unhelpful’). Qualitative descriptors of effect sizes provided by Rosenthal [50] were used to interpret the differences between the ‘helpful’/‘unhelpful’ ratings of the undergraduates and experts (i.e., the difference in the proportion of undergraduates and experts rating each of these options as either ‘helpful’ or ‘unhelpful’). Differences of ≥18 and ≥30 indicated at least a medium and large effect size respectively.

### Coding of question relating to help-seeking intentions

This open-ended question was coded by the first author (SDA), a clinical psychologist trained in Sri Lanka and fluent in English and Sinhala, the languages used by most participants. English translations for the Tamil responses were provided by a professional translator. Coding categories used in similar studies provided preliminary guidance for coding [13, 37]. All responses that differed in meaning were coded as separate categories. If responses were relevant to multiple coding categories, they were coded as such. Subsequent to this, the authors re-grouped these categories into broader coding categories.

### Ethics approval

Approval for all components of the study was obtained from the Ethics Review Committees of the Faculty of Medicine, University of Colombo, and University of Melbourne.

### Statistical analysis

Binary logistic regression models were used to examine if the medical undergraduates differed from the non-medical undergraduates (*IV*) in their odds of: (1) having a PHQ-9 diagnosis of Major Depression (*DV*) [51]; (2) rating the help-seeking options as ‘helpful’/‘unhelpful’ (*DV*); and (3) intending to seek help from the commonly nominated sources if affected by the problem presented in the vignette (*DV*). The non-medical undergraduate group was the reference category for these analyses.

Binary logistic regression models were also used to examine if within the medical undergraduate group, those screening positive for Major Depression differed from those screening negative (*IV*) in their odds of: (4) rating the help-seeking options as ‘helpful’/‘unhelpful’ (*DV*); and (5) intending to seek help from the commonly nominated sources if affected by the problem presented in the vignette (*DV*). Those screening negative for Major Depression were the reference category for these analyses.

The following demographic variables were adjusted for in each of these analyses: gender, year of study, age category, religion and residence. The language used for indicating help-seeking intentions was adjusted for in analyses 3 and 5.

## Results

Rough head-count estimates done at the administration sites were compared with the number of questionnaires that were returned by the participants and this indicated that the participation rate was very high (approaching 100 %). Responses were obtained from 620 medical and 4050 non-medical undergraduates (Arts and Education: *n* = 1198; Law: *n* = 616; Management and Finance: *n* = 1025; Science: *n* = 687; School of Computing: *n* = 524). These samples were approximately 60 % of the medical undergraduates and 52 % of the non-medical undergraduates enrolled at the University of Colombo. The demographic and other characteristics of the two groups are presented in Table 1.

**Table 1:** Demographic and other characteristics of undergraduates (UGs)

Variable	Medical UGs (*n* = 620)		Non-medical UGs (*n* = 4050)	
	f	%	f	%
Demographic variables				
Gender				
Male	267	43.1	1180	29.1
Female	352	56.8	2867	70.8
Year of study				
1st year	144	23.2	1802	44.5
2nd year	122	19.7	1120	27.7
3rd year	124	20.0	714	17.6
4th year	116	18.7	414	10.2
5th year	114	18.4	NA	NA
Age category				
18–20 years	34	5.5	481	11.9
21–23 years	352	56.8	3003	74.1
24 and above	233	37.6	559	13.8
Residence				
Home	170	27.4	1582	39.1
Hostel	263	42.4	1139	28.1
Rented place	155	25.0	1033	25.5
Home of friend/relative	9	1.5	263	6.5
Other	22	3.5	29	0.7
Religion				
Buddhist	518	83.5	3545	87.5
Hindu	50	8.1	111	2.7
Islamic	14	2.3	138	3.4
Roman Catholic	27	4.4	188	4.6
Other	9	1.5	64	1.6
Other variables				
Screening positive for Major Depression^a^	58	10.2	343	9.2

The percentages for variable categories do not sum to a total of 100 % due to missing responses

^a^The percentages have been calculated in relation to the valid responses for the PHQ-9 (only 1 missing item permitted) (medical UGs, *n* = 567; non-medical UGs, *n* = 3736)

### (1) Comparison of prevalence of depression among medical and non-medical undergraduates

A previous analysis of data from the current samples found that 9.3 % of undergraduates screened positive for Major Depression on the PHQ-9 [52]. The present analysis found that the odds of screening positive for Major Depression did not differ between the medical and non-medical undergraduates [*OR* = 1.04; 95 % *CI* (0.74, 1.47); *p* = .81].

### (2) Comparison of help-seeking perceptions of medical and non-medical undergraduates

As compared to the non-medical undergraduates, the medical undergraduates had higher odds of rating the following options as ‘helpful’: psychologist, university student counsellor, university medical officer, “western medicine to improve mood” and talking to others who have faced similar problems (Table 2).

**Table 2:** A comparison of the help-seeking perceptions of medical and non-medical undergraduates (UGs), using logistic regression

Help-seeking option	Option rated as ‘helpful’/‘unhelpful’			Odds of medical UGs giving rating (compared with non-medical UGs)	
	% of Experts	% of Medical UGs (*n* = 606-618)	% of Non-medical UGs (*n* = 3895-4033)		
				Adjusted odds ratio [95 % CI] (*n* = 4480-4629)	
Recommended professional/formal options: rated as ‘helpful’ by experts					
Psychiatrist	100	89.7	88.8	1.12	[0.82, 1.52]
Psychologist	100	81.2^b^	64.4^a^	2.09^***^	[1.64, 2.65]
Counsellor	89.2	90.9	90.7	1.18	[0.84, 1.65]
Organisation helping people to deal with problems	83.3	51.3^a^	48.3^a^	1.01	[0.83, 1.22]
Mental health professional at university psychiatry unit	100	69.3^a^	70.7^b^	1.01	[0.82, 1.25]
University medical officer	88.9	51.8^a^	50.9^a^	1.24^*^	[1.02, 1.50]
University student counsellor	75.0	78.0	74.9	1.66^***^	[1.30, 2.12]
Western medicine to improve mood	97.1	49.2^a^	25.9^a^	2.40^***^	[1.97, 2.92]
Get counselling/psychological therapy	100	88.6	88.5	1.17	[0.86, 1.58]
Recommended informal options: rated as ‘helpful’ by experts					
Friend from university	88.6	91.3	91.7	1.03	[0.73, 1.46]
Parents	78.4	93.5	92.9	1.35	[0.91, 2.00]
Boyfriend/girlfriend/spouse	83.8	88.9	87.5	0.90	[0.68, 1.21]
Talk to others who have faced similar problems	85.7	84.5	75.3	1.73^***^	[1.34, 2.23]
Options identified as ‘unhelpful’ by experts					
Deal with problem alone	86.1	59.7^b^	63.2^b^	1.02	[0.84, 1,25]

*p < .05; ***p < .001

Expert opinion has been used as a benchmark for assessing help-seeking perceptions of undergraduates

^a^where the difference in ratings of UGs and mental health experts had a large effect size (≥30 %)

^b^where the difference in ratings of UGs and mental health experts had a medium effect size (≥18 %)

When using the opinions of mental health professionals about help-seeking as the benchmark for comparison (Table 2), the related ratings of the medical undergraduates were lower, differing with medium to large effect sizes, in the case of the following options: psychologist, organisation helping people to deal with problems, mental health professional at university psychiatry unit, university medical officer, “taking western medicine to improve mood” and dealing with the problem alone (the latter being the only instance an ‘unhelpful’ rating was relevant). However, the ‘helpful’/‘unhelpful’ ratings of the non-medical undergraduates also deviated from expert opinion in relation to these options.

### (3) Comparison of help-seeking intentions of medical and non-medical undergraduates

Given the lower number of respondents in the medical undergraduate group, only sources that approximately 7 % or more of this group nominated (>40 of respondents) were considered for analysis (Table 3). Informal sources such as friends and parents were nominated more than the professional sources by both the medical and non-medical undergraduates. Although psychiatrists were the professional help-provider most nominated by the medical undergraduates, they were nominated by only 10.5 %. As seen in Table 3, compared to the non-medical undergraduates, the medical undergraduates had higher odds of intending to seek help from parents and family members. There were no other differences in the help-seeking intentions of these two groups.

**Table 3:** A comparison of the help-seeking intentions of medical and non-medical undergraduates (UGs), using logistic regression

Help-seeking source	Intentions to seek help		Odds of medical UGs intending to seek help (compared with non-medical UGs)	
	% of Medical UGs (*n* = 581)	% of non-medical UGs (*n* = 3879)		
			Adjusted odds ratio [95 % CI] (*n* = 4442)	
Professional help				
Psychiatrist	10.5	6.9	1.23	[0.78, 1.93]
Doctor/medical assistance/medical treatment	6.7	7.8	0.77	[0.51, 1.16]
Counsellor/counselling	7.7	6.3	1.24	[0.82, 1.85]
Informal help				
Friend	38.0	33.6	1.24	[1.00, 1.55]
Parent	23.1	19.7	1.33^*^	[1.03, 1.72]
Family member/s	7.6	4.4	1.68^*^	[1.10, 2.56]
Getting help but person not specified^a^	10.5	14.4	0.96	[0.69, 1.35]

*p < .05

^a^As the help-provider was not specified these responses were considered within the category of informal help

### (4) Comparison of help-seeking perceptions of medical undergraduates screening positive/negative for Major Depression

As compared to those screening negative for Major Depression, those who screened positive had lower odds of endorsing the following recommended options as being ‘helpful’: psychiatrist, counsellor, university student counsellor, friend from university, parents and boyfriend/girlfriend/spouse (Table 4).

As seen in Table 4, when using the mental health professionals’ opinions as the benchmark for comparison, the ‘helpful’ ratings given by those screening positive for Major Depression deviated to a greater extent than the ratings of those screening negative (five of the eight options for which medium or large effect sizes were observed). The ‘unhelpful’ rating given by those screening positive for Major Depression, for the option of dealing with the problem alone, differed from expert opinion with a large effect size. Comparatively, the rating of those screening negative only deviated with a medium effect size. Furthermore, only 50 % of those screening positive for Major Depression rated this option as ‘unhelpful’.

**Table 4:** A comparison of the help-seeking perceptions of medical undergraduates (UGs) screening positive/negative for Major Depression, using logistic regression

Help-seeking options	Option rated as ‘helpful’/‘unhelpful’			Odds of those screening positive giving rating (compared with those screening negative)	
	% of Experts	% of those Screening positive for Major Depression (*n* = 57-58)	% of those screening negative for Major Depression (*n* = 498-509)		
				Adjusted odds ratio [95 % CI] (*n* = 553- 565)	
Recommended professional/formal options: rated as ‘helpful’ by experts					
Psychiatrist	100	79.3^b^	91.3	0.28^**^	[0.13, 0.62]
Psychologist	100	77.2^b^	82.3	0.73	[0.36, 1.48]
Counsellor	89.2	81.0	92.7	0.31^**^	[0.14, 0.70]
Organisation helping people to deal with problems	83.3	51.7^a^	52.2^a^	1.00	[0.56, 1.77]
Mental health professional at university psychiatry unit	100	61.4^a^	72.1^b^	0.65	[0.36, 1.18]
University medical officer	88.9	41.4^a^	54.1^a^	0.60	[0.34, 1.07]
University student counsellor	75.0	65.5	80.4	0.51^*^	[0.28, 0.96]
Take western medicine to improve mood	97.1	50.0^a^	49.7^a^	0.99	[0.55, 1.79]
Get counselling/psychological therapy	100	81.0^b^	90.2	0.49	[0.22, 1.08]
Recommended informal options: rated as ‘helpful’ by experts					
Friend from university	88.6	79.3	92.9	0.31^**^	[0.14, 0.67]
Parents	78.4	86.0	94.1	0.34^*^	[0.14, 0.82]
Boyfriend/girlfriend/spouse	83.8	78.9	90.3	0.37^**^	[0.17, 0.78]
Talk to others who have faced similar problems	85.7	91.4	84.4	2.35	[0.81, 6.79]
Options identified as ‘unhelpful’ by experts					
Deal with problem alone	86.1	50.0^a^	61.3^b^	0.68	[0.39, 1.21]

*p < .05; **p < .01

Expert opinion has been used as a benchmark for assessing help-seeking perceptions of undergraduates

^a^where the difference in ratings of UGs and mental health experts had a large effect size (≥30 %)

^b^where the difference in ratings of UGs and mental health experts had a medium effect size (≥18 %)

### (5) Comparison of Help-seeking intentions of medical undergraduates screening positive and negative for Major Depression

Those screening positive and negative for Major Depression did not differ significantly in their help-seeking intentions and hence, the related results are not reported.

## Discussion

This study examined whether medical undergraduates differed from their non-medical peers in relation to their prevalence of depression and perceptions and intentions relating to help-seeking. We also examined whether depression was associated with differences in the help-seeking perceptions and intentions of medical undergraduates. The findings indicate that medical and non-medical undergraduates are at similar risk of being depressed. Although medical undergraduates were more likely to appraise professional help positively, their personal intentions about seeking such help did not differ from those of non-medical undergraduates. Furthermore, being depressed was not associated with changes in their help-seeking intentions and in fact was associated with less positive appraisal of recommended options of help.

Although it is of concern that one tenth of the medical undergraduates screened positive for Major Depression, the findings indicate that being a medical undergraduate might not be associated with a higher risk of being depressed and that instead, high rates of depression might be a common issue faced across the undergraduate population. Hence, as discussed in Amarasuriya *et al.* [52], it is necessary to develop response mechanisms to assist all of these undergraduates.

The findings indicate that the medical undergraduates’ appraisal of some of the help-seeking options, such as the use of *western medicine to improve mood*, did not always align with expert opinion, indicating the need for interventions to improve their attitudes towards these to bolster their related help-seeking. Nevertheless, when compared to their non-medical counterparts, they were more likely to endorse some of the professional help-seeking options indicating their greater knowledge about dealing with depression.

Despite this, only a low proportion of medical undergraduates indicated their personal intentions to seek professional help if affected by the problem, with their help-seeking intentions not differing from those of their non-medical counterparts. Instead, they were more likely to indicate that they would seek help from their parents and family, concurring with previous studies showing a preference among medical undergraduates to seek help from informal sources [18, 20–23].

The findings indicate that although medical undergraduates might be knowledgeable about options for professional help and have favourable attitudes towards many of these options, such knowledge and attitudes might not be reflected in their attempts to deal with their own problems. Hence, there might be dissonance between their treatment recommendations for their patients and their self-care practices, pointing to the need for curriculum elements to address this. It is also important to simultaneously educate the informal help-providers that these medical undergraduates approach about providing mental health first-aid [53]. Given that the help of friends has been highly endorsed by them, it might be beneficial to provide medical undergraduates with mental health first-aid training to improve their related skills and decrease their stigmatising attitudes about their mentally ill peers [54].

The findings indicate that being depressed might not have any effect on these medical undergraduates’ intentions to seek help. Moreover, the treatment beliefs of those with depression might differ considerably from expert opinion with regard to the use of professional help for depression. Comparison of the perceptions of those screening positive and negative for Major Depression about the *helpfulness* of the recommended options, indicate that regardless of whether these options are professional or informal, being depressed might be associated with less positive appraisal of these among the medical undergraduates. This warns of the effects that such appraisals might have on the actual help-seeking behaviours of this group of undergraduates. As compared to those who were not depressed, those who screened positive for Major Depression were less aware that it was unhelpful to try and deal with the problem alone. This finding is especially concerning, as 50 % of this group did not consider that dealing with the problem alone was ‘unhelpful’, and as this provides further warning that there could be delays in their initiation of seeking any type of help for their depression, be it from professional or informal sources.

While the findings indicate that these medical undergraduates might be reluctant to seek help for their depression, they point to the need for examining the reasons for this. It is necessary to examine whether such reluctance is associated with their *perceived* negative consequences of seeking help, such as a lack of confidentiality, academic and career impact and discrimination, especially given the small network of mental health professionals in the country. However, in light of the findings relating to the medical undergraduates’ appraisals of the different options of help, it is also important to determine if instead, such findings are related to their actual negative experiences of seeking help [18].

Although it might not be feasible at present to develop specialised mental health services for these medical undergraduates, given the limited number of mental health professionals in the country, it is necessary to understand if any mechanisms that are currently available could be developed. However, our findings indicate that this could be a complex task given these undergraduates’ appraisal of the available services. The low ‘helpful’ ratings given by the medical undergraduates (compared to experts) to mental health professionals from the university psychiatry unit and the university medical officer might be associated with their reluctance to approach university-related personnel. Other studies have found that medical undergraduates are reluctant to even disclose the mental health problems of peers to medical school administration [55]. However, the findings were similar among the non-medical undergraduates. Nevertheless, the findings indicate that these medical undergraduates’ rating of student counsellors is more in line with that of experts, indicating the usefulness of such personnel as contact points for distressed undergraduates. However, our findings also indicate that those who are depressed might be less likely to consider these help-providers as being ‘helpful’. This further highlights the need to identify pathways of help for these medical undergraduates and to examine the potential reasons for their reluctance to seek help; especially among those who are distressed.

The findings must be considered in light of the limitations of the study. It must be noted that this study only examined help-seeking perceptions and intentions, and that actual practice might differ. Furthermore, the findings for the help-seeking intentions question might have been different if fixed rating scale items had been presented instead of open-ended questions. However, it is argued that the study methodology is more reflective of the cognitive mechanisms that this population would undergo in deciding the type of help to seek, if affected by depression. Given that this study employed a vignette as the stimulus for responses of participants, and as this methodology might not always be able to capture real life manifestations of depression, it is necessary to consider whether the responses of the medical undergraduates were limited by the lack of such descriptive detail in the vignette. Although administering the questionnaire during lectures ensured a high response rate, the exclusion of those who did not attend lectures from the study sample might have led to a loss of data from those whose absence was due to mental health problems that they were facing. Further extension of this work among those experiencing other common disorders found among undergraduate populations, such as Anxiety Disorders, would provide a greater understanding of mental health help-seeking among this population.

## Conclusions

Medical and non-medical undergraduates are at similar risk of being depressed. Overall, compared to non-medical undergraduates, medical undergraduates are more likely to have greater knowledge and favourable attitudes about the use of professional help for depression. However, their personal intentions to seek such help are low, not differing from those of their non-medical peers. Instead, they show higher intentions of seeking help from informal sources such as, parents and family. Furthermore, it is concerning that depressed medical undergraduates might have inadequate help-seeking practices, as a considerable proportion do not consider it ‘unhelpful’ to deal with the problem alone, as their intentions to seek help do not differ from those who are not depressed, and as compared to the latter group, they are less likely to positively appraise some of the formal and informal sources of help recommended for dealing with depression.

## Additional files

Additional file 1: **English-Sinhala version of questionnaire.** (PDF 189 kb) Additional file 2: **Items identified as ‘helpful’ and ‘unhelpful’ by ≥ 75 % of experts.** (PDF 183 kb)

## Abbreviations

IV: Independent variable
DV: Dependent variable
PHQ-9: Patient Health Questionnaire-9
Undergraduate: UG (only used in Tables)
