# Supplementary material for: Perceptions and intentions relating to seeking help for depression among medical undergraduates in Sri Lanka: a cross-sectional comparison with non-medical undergraduates
Source: BMC Med Educ. 2015 Sep 29;15:162. doi: 10.1186/s12909-015-0453-8 (PMC4589186; doi:10.1186/s12909-015-0453-8)
Supplement: Additional file 2: — Items identified as ‘helpful’ and ‘unhelpful’ by ≥ 75 % of experts. (PDF 183 kb) [file 12909_2015_453_MOESM2_ESM.pdf]

Items identified as 'helpful' and 'unhelpful' by ≥ 75% of mental health experts

Rated as 'helpful' by ≥ 75% of mental health experts

Professional/ formal

- Psychiatrist *
- Psychologist *
- Counsellor *
- Organisation helping people to deal with their problems *
- A mental health professional at the University Psychiatry Unit *
- A university student counsellor *
- A university medical officer *
- Take western medicine to improve mood *
- Get counselling or psychological therapy *

Informal (including self-help strategies)

- Parents *
- Friend from University *
- Boyfriend/ Girlfriend/ Spouse *
- Become more active in daily activities
- Do physical exercise
- Do activities he/ she enjoys
- Do meditation, yoga or other relaxation exercises
- Improve sleeping habits
- Get information from internet about dealing with problem
- Talk to others who have faced similar problems *
- Cut down use of alcohol/ cigarettes/ drugs

Rated as 'unhelpful' by ≥ 75% of mental health experts

- Not approach anyone for help and deal with problem alone *
- Stop going to university and stay at home
- Use alcohol/ cigarettes/ drugs

* Options with involve the assistance of others
